# Maryland State Veterinary Medical Society

**Published:** 1891-04

**Authors:** H. A. Meisner

**Affiliations:** Secretary


*Maryland State Veterinary Medical Society.*—The fifth annual and forty-ninth regular meeting of the Maryland State Veterinary Medical Society was held February 19th, Madison Avenue and Orchard Street, Baltimore, with the President, Dr. W. H. Martenet in the chair. Roll was called to which the following members responded: Drs. W. H. Martenet, Geo. C. Faville, Wm. Dougherty, T. F. Barron, and H. A. Meisner. The minutes of the previous meeting were read and approved. Dr. Dougherty reported that the U. S. Veterinary Medical Association would hold their next annual meeting in Washington during September. Committee discharged with thanks. Report of Secretary and Treasurer was read and approved.

Under the head of new business Dr. Dougherty moved that a Committee of three be appointed to correspond with the Veterinarians of Washington relative to entertaining the U. S. Veterinary Medical Association. Motion carried and Drs. Dougherty, Faville, and Meisner were appointed. A paper on "The Duties and Responsibilities of a Veterinarian" by Dr. A. W. Clement, who was unable to be present was read with much credit by Dr. Geo. C. Faville. Dr. Dougherty was appointed essayist for the next meeting. Election of officers: Secretary was ordered to cast a ballot for

Dr. W. H. Martenet, for President, and Dr. Wm. Dougherty, Vice President, Dr. H. A. Meisner was elected Secretary and Treasurer. Board of Censors for the ensuing year are Drs. Wm. Dougherty, T. F. Barron, A. W. Clement, D. R. Hoffman and E. C. Schroeder.

After the election of officers the meeting was adjourned to meet at Montgomery's Hotel, where their annual banquet was held, during which a communication from Dr. Clement on Pseudo-Tuberculosis was read by Dr. Faville.

H. A. MEISNER, V. M. D., *Secretary.*

